# Aging of severely mentally ill patients first admitted before or after the reorganization of psychiatric care in Sweden

**DOI:** 10.1186/s13033-022-00544-9

**Published:** 2022-07-13

**Authors:** Pia H. Bülow, Deborah Finkel, Monika Allgurin, Cristina Joy Torgé, Magnus Jegermalm, Marie Ernsth-Bravell, Per Bülow

**Affiliations:** 1 Department of Social Work, School of Health and Welfare, Jönköping University, Jönköping, Sweden; 2 Research Fellow in Department of Social Work, University of the Free State, Bloemfontein, South Africa; 3 Institute of Gerontology, School of Health and Welfare, Jönköping University, Jönköping, Sweden; 4 Department of Psychology, Indiana University Southeast, New Albany, USA; 5 Institute of Gerontology, School of Health and Welfare, Jönköping University, Jönköping, Sweden; 6 Department of Social Work, School of Health and Welfare, Jönköping University, Jönköping, Sweden; 7 Regional Forensic Psychiatric Hospital, Vadstena, Sweden

**Keywords:** Older people, Severe mental illness, Deinstitutionalization, Sectorization, Longitudinal

## Abstract

**Background:**

The concept of deinstitutionalization started in the 1960s in the US to describe closing down or reducing the number of beds in mental hospitals. The same process has been going on in many countries but with different names and in various forms. In Europe, countries like Italy prescribed by law an immediate ban on admitting patients to mental hospitals while in some other European countries psychiatric care was reorganized into a sectorized psychiatry characterized by open psychiatric care. This sectorization has not been studied to the same extent as the radical closures of mental hospitals, even though it entailed major changes in the organization of care. The deinstitutionalization in Sweden is connected to the sectorization of psychiatric care, a protracted process taking years to implement.

**Methods:**

Older people, with their first admission to psychiatric care before or after the sectorization process, were followed using three different time metrics: (a) year of first entry into a mental hospital, (b) total years of institutionalization, and (c) changes resulting from aging. Data from surveys in 1996, 2001, 2006, and 2011 were used, together with National registers.

**Results:**

Examination of date of first institutionalization and length of stay indicates a clear break in 1985, the year when the sectorization was completed in the studied municipality. The results show that the two groups, despite belonging to the same age group (birthyears 1910–1951, mean birthyear 1937), represented two different patient generations. The pre-sectorization group was institutionalized at an earlier age and accumulated more time in institutions than the post-sectorization group. Compared to the post-sectorization group, the pre-sectorization group were found to be disadvantaged in that their level of functioning was lower, and they had more unmet needs, even when diagnosis was taken into account.

**Conclusions:**

Sectorization is an important divide which explains differences in two groups of the same age but with different institutional history: “modern” and “traditional” patient generations that received radically different types of care. The results indicate that the sectorization of psychiatric care might be as important as the Mental Health Care Reform of 1995, although a relatively quiet revolution.

## Background

The deinstitutionalization of psychiatric care, that is the closing down or reducing the number of beds in mental hospitals, started in the 1960s in the US but the same process has been going on in many countries. Since then, many studies have been conducted to investigate the impact of this change. One of the most comprehensive studies was known as the TAPS-project (Team for the Assessment of Psychiatric Services) [1], which followed the closing down of two mental hospitals in London showing a successful transition to small community-based residential care. Most studies, however, have criticized deinstitutionalization arguing that people with severe mental illness (SMI) have been abandoned [2] and earlier follow-up studies reported that people who previously lived in mental hospitals became homeless [3], ended up in prisons [4], died prematurely and committed suicide to a greater extent compared to the situation before this change [5]. Critics argue that such negative consequences are mainly due to a lack of alternative forms of care and support [6]. However, to our knowledge, there are no studies of the impact of deinstitutionalization which take into account time in institution prior to changes in care. Moreover, few studies highlight the length of time living with SMI, and the meaning of aging with SMI (authors). Existing studies typically have at most five years of follow-up [7]. Furthermore, most studies of older adults who experienced institutionalization focus on dementia disorders, instead of severe mental illness. The trends for secular changes in the epidemiology of mental disorders in Sweden, in the past decades, show an increase of especially younger adults utilizing any mental health care [8]. The group of people with SMI is however relatively stable. In this article, we discuss deinstitutionalization by comparing the outcomes for two groups of older people with severe mental illness (SMI) in the same age range but with different experiences of inpatient care who were followed up for 15 years.

### Swedish model of “deinstitutionalization”

Deinstitutionalization in Sweden took the form of sectorization of the psychiatric care [9]. This reorganization implied that each municipality was divided into geographical sections and that each section was responsible for inpatient as well as outpatient psychiatric care. However, in an experimental study, Lindholm [10] concluded “that there are no consistent tendencies proving the new organisation superior to the traditional one for these former mental hospital patients” (p. 88). In a later study, Stefansson and Hansson [11] noted that the group of people with SMI in Sweden did not benefit from the outpatient care as part of this reorganization. However, to our knowledge, it has never been demonstrated which groups have been most affected: the SMI-patients already identified as long-stay patients who did not benefit from the sectorized organisation of psychiatric care, or the whole group of patients with SMI who were neglected by the open care teams.

### Sectorization—the quiet revolution

The concept of deinstitutionalization in Europe does not have a simple and agreed upon meaning. There is a vast difference between radical changes as in Italy, where Law 180 from 1978 entailed an immediate ban on admitting patients to mental hospitals [12] to the rather slow process of reorganizing psychiatric care into a sectorized psychiatry characterized by open psychiatric care, which developed gradually, especially in Europe. This sectorization has not been studied to the same extent as the radical closures of mental hospitals, despite the fact that it entailed major changes in how care was organized. That sectorization has been relatively "invisible" in research can be explained by the fact that this reorganization of psychiatric care in many countries was a protracted process that took decades to implement. This prompted Gittelman [9] to designate it the "quiet revolution".

The reorganization of psychiatric care following the sectorization principle was established in many European countries, but at different times. In the Netherlands, this reorganization of psychiatric care started in the 1940s, in the aftermath of World War II, as an alternative to the cost of rebuilding the mental hospitals demolished by war [13]. This process took several years to complete. In France, where the term sectorization was first coined in the 1960s [14], the process also took many years. This delay was partly due to a dispute between different groups of psychiatrists about the role of mental hospitals in care and social rehabilitation, where sectorization became a compromise between the two perspectives [15]. The compromise was the creation of different areas, so-called sectors (in French: secteurs) which were all linked to a mental hospital.

In Sweden, the reorganization of psychiatric care started late in comparison with other European countries such as U.K., France, and the Netherlands. The reason for this was, among other things, that the mental hospitals received large government grants and that many of them were located in smaller towns and created many local jobs. However, psychiatric care organization was subjected to investigations during the 1970s and the National Board of Health and Welfare (in Swedish: Socialstyrelsen) criticized the mental hospitals, pointing out that patients were apathetic, lacking initiative, and unable to plan for the future, as a consequence of long stays in hospitals ([16], p. 23).

In reaction to this criticism National Board of Health and Welfare, advocated that psychiatry should be organized according to the sectorization principle [17]. During the 1970s, this form of organization was relatively unusual in Sweden and initially started as a trial project. First out was the Nacka project, which started in 1974 [13]. As in the Netherlands, it was the insufficient number of beds in psychiatric care that forced an outpatient-based alternative in Nacka and Värmdö, two municipalities in Stockholm County. The purpose of organizing care according to the sectorization principle was, in Sweden, to reduce the number of admissions to the hospital through open care, shorter time spent in hospitals and to prevent readmissions. In an experimental study from 1983, Lindholm studied the outcome regarding satisfaction with care, social function level, burden on relatives, consumption of psychiatric care and costs. Lindholm [9] stated that the outcome did not differ between the experimental group that received care according to the sectorization principle and the control group that received traditional care.

### The continuation of reorganizing psychiatric care

Sectorized psychiatry in Sweden resulted in shorter in-patient periods and the number of hospital beds decreased, but above all a new group of people who previously did not have access to psychiatric care and did not need hospitalizations, was given access to psychiatric care. These were people with individual psychological problems such as anxiety, sleeping disorders and relationship problems, a group that in the literature is called "the worried well" [10, 18]. On the other hand, people with SMI did not benefit from the more open and society-based psychiatry and thereby did not benefit from sectorized psychiatry [11]. People with SMI, especially those with a schizophrenia diagnosis, were still largely cared for in institutions, which was due to the lack of adequate alternatives for that group. Regular national surveys had been conducted to follow the living conditions of the Swedish population. In the 1988/1989 survey of living conditions, (in Swedish: Undersökning av Levnadsförhållanden, ULF-F) by Statistics Sweden, it was found that people with SMI had living conditions far below the average for the Swedish population and lower than groups with physical disabilities as well [19]. This prompted the Swedish Parliament to appoint an inquiry (called the Psychiatric inquiry) with the task of proposing measures to improve the situation for people with SMI. The inquiry resulted in a report published in 1992 [20]. The Community Mental Health reform clarified the responsibilities of social services and psychiatry respectively, for people with SMI. The clarification meant that the municipalities became responsible for providing support to persons with SMI regarding housing, employment, and an active everyday life, thereby establishing conditions to integrate into society. The county council's responsibility was to treat and prevent mental illness. The reform emphasised that social services and psychiatry must cooperate to prevent people from “falling between the cracks”. Some government funding accompanied the reform, which came into force in 1995. The reorganization of psychiatric care in Swedish can thus be described as divided into two phases, sectorization (in principle implemented throughout Sweden in 1984) and the implementation of the Mental Health Care Reform in 1995. The reduced number of beds began at the time of the sectorization or shortly before, but the sectorization meant it became possible to treat people in outpatient care in the communities.

In this article, we discuss the reorganization of psychiatric care by examining two groups of older people in the same age range (birthyears 1910–1951) who had their first admission to psychiatric care before or after the sectorization process was completed. In the municipality where this study was conducted, the county council decided in 1979 to reorganize psychiatric care according to the principles of sectorization; the process began in 1980 and was completed in 1984. During these years, the number of beds in the local mental hospital was reduced from 809 in 1979 to 489 in January 1985. The aim of the study is to investigate functioning and needs for older people experiencing institutionalization before or after sectorization using three different time metrics: (a) year of first entry into a mental hospital, (b) total years of institutionalization, and (c) changes resulting from aging with SMI.

## Methods

In this study we used data from surveys repeated 4 times covering 15 years of follow-up of living conditions, needs and functioning, of older adults with SMI, combined with national registers covering decades of in-patient care.

### Data and participants

At the outset of the Mental Health Care Reform in 1995, National Board of Health and Welfare directed all municipalities and county councils in Sweden to identify the number of persons over the age of 18 assessed as SMI and what needs this group had regarding care and support. In Jönköping, a medium-sized municipality in southern Sweden, such surveys were conducted every fifth year, starting in 1996 [21, 22]. Our project focused on following older people with SMI, with the sample of 653 people who in 2016 were 65 years or older: severely mental ill-older (SMI-O) [23]. Data were collected from the surveys from the years 1996, 2001, 2006, and 2011. The surveys were conducted by mental health care staff from the county council and social workers from the municipality and included staff assessments of clients as well as client ratings of their own needs. The staffs were jointly trained to use the assessment instruments in a similar way. To reach the entire target group, primary care, employment services, voluntary organizations, the Swedish Social Insurance Agency and the police were contacted. In combination with information associated with the personal identification number, this approach resulted in over 90% of the target group being included at each wave either by interviews and staff assessments, or only the latter (personal communication, 29 April 2020).

In addition, based on the survey participants’ personal identification numbers, data were drawn from National registers; Statistics Sweden, Cause of death register and National Patient Register. The current study was approved by the Regional Ethical Review Board in Linköping, Sweden. For the current analyses, the SMI-O group was divided into two groups based on the date of their first entry into a mental hospital: pre and post sectorization groups.

### Measures

Information about institutionalization. Data from the National Patient Register allowed us to calculate several variables describing or summarizing information about inpatient care. First, the date of the first institutionalization was available. Second, we used birthdate to calculate age at first institutionalization. Third, the registry included information about the length of each stay in an institution, so we were able to calculate the number of stays, the total years of institutionalization, and then the average length of stay. Total years of institutionalization was first calculated in number of days, which was then translated to years by dividing total number of days by 365.25.

#### The National Board of Health and Welfare’s Inventory form (NBHWI)

Nine items from the National Board of Health and Welfare's Inventory form (NBHWI) assessing functioning were combined to create a daily functioning measure: hygiene, household, food preparation, finances, telephone, daily activity, travel to another county, travel to another city, and contact with authorities [24]. Each item was rated by the interviewer on a scale from 1 (manage without help) to 3 (cannot manage), so higher scores indicate more functional difficulties. This measure was not included in the 2011 interview.

#### General Assessment of Functioning (GAF)

The Global Assessment of Functioning (GAF) is a measure of symptom and social disability. GAF is a numeric scale to rate subjectively the social, occupational, and psychological functioning of an individual [25]. Scores range from 100 (extremely high functioning) to 1 (severely impaired), so higher scores indicate better functioning. Interrater reliability for the GAF has been estimated at 0.89 and it has shown strong concurrent validity [26].

#### Camberwell Assessment of Need (CAN)

Camberwell Assessment of Need (CAN) assesses needs in 22 different areas of life and estimates the degree of satisfaction within each area [27]. The data can be combined to result in four summary variables: Total number of needs identified (Needs), Points (indicating…), Number of “1”ratings (moderate problems), and Number of “2” ratings (severe problems). For each area it is possible to identify personal problems, and if people have help to deal with these problems and if they are satisfied with the help given. The CAN assessments includes both general human needs and needs specific to people with SMI; higher scores indicate more difficulties, needs or problems. Interrater reliability for the CAN has been estimated at 0.85 and higher, and it demonstrates strong construct validity [28].

### Statistical analysis

Two analytical approaches were used to investigate the impact of the reorganization of psychiatric care on functioning: categorical and continuous. For categorical analyses, chi-square tests and independent samples t-tests were used to compare the pre-sectorization and post-sectorization groups on outcome variables. The continuous analyses incorporated the three different time metrics: year of first entry into a mental hospital, total years of institutionalization, and age of first participation in the Jönköping surveys of SMI-O were included as continuous variables in linear regression analyses to predict functioning outcomes. The predictive value of these variables was examined in the context of two other relevant variables: sex and whether or not the person had been diagnosed with a psychosis (0 = no, 1 = yes).

## Results

### Pre- and post-sectorization groups

**Fig. 1:**
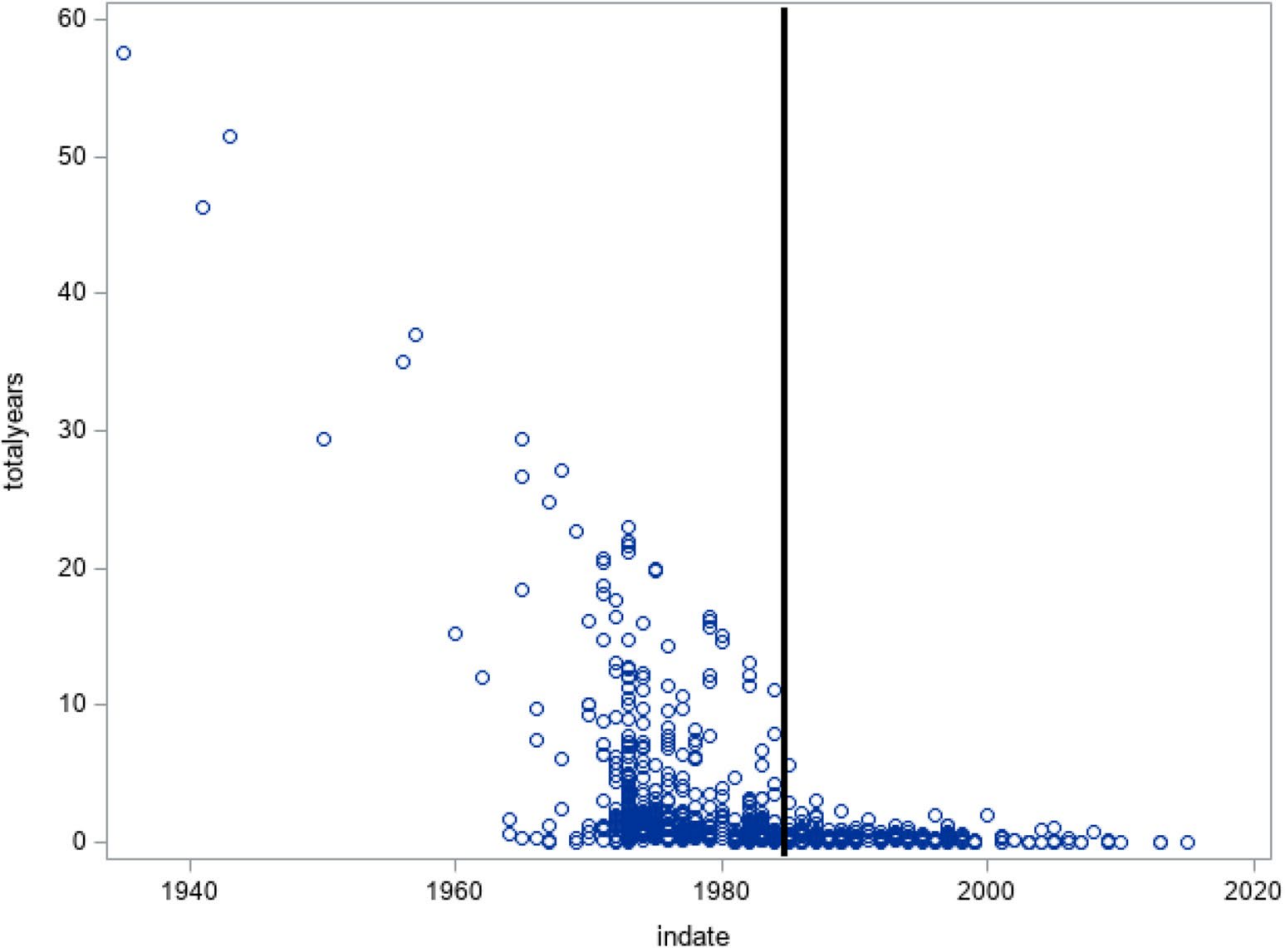
Scatterplot of total years of institutionalization (y-axis) and year of first institutionalization (x-axis). Vertical line indicates end of organizational changes (sectorization) in mental health care (1985)

Examination of date of first institutionalization and length of stay in the SMI-O sample indicates a clear break in 1985, the year when the sectorization was completed in the studied municipality. As shown in Fig. 1, no one first admitted after 1985 accumulated more than 6 years in a mental hospital. Individuals with accumulated time in a mental hospital of 10 years or more all had a first date of entry prior to 1985. Therefore, the SMI-O sample was divided into a pre-sectorization group with first entry prior to January 1, 1985, and a post-sectorization group with first entry on or after January 1, 1985. As shown in Table 1, sample sizes varied across surveys based on participation rates, and over time 44% of the sample died.

**Table 1:** Sample sizes at each survey year for pre-reform and post-reform groups

Survey year	1996	2001	2006	2011
Pre-reform group				
Participated	277	272	182	158
Did not participate	174	127	138	97
Died before survey^a^	–	52	131	196
Total	451	451	451	451
Post-reform group				
Participated	74	86	71	75
Did not participate	124	100	89	59
Died before survey	–	12	38	64
Total	198	198	198	198

^a^Died before Survey indicates cumulative number of deaths to that year, i.e., an additional 79 people in the pre-reform group died between 2001 and 2006 for a total number of deaths prior to the 2006 survey of 131

Sample characteristics for the pre- and post-sectorization groups are presented in Table 2. The two groups did not differ in mean birth year (1937), range in birth years (1910–1951), education categories measured on a scale from 1 (less than 9 years) to 7 (postgraduate education), or age of first participation in the SMI-O surveys. There were significantly more women in the post-sectorization group than pre-sectorization. Although over half of the pre-sectorization group had a diagnosis of psychosis, only one-third of the post-sectorization group did. In contrast, the post-sectorization group was more likely to have a diagnosis of neurosis (F40-F48 in ICD-10, 1992) than the pre-sectorization group (26% vs. 12%). Diagnosis rates in other categories (addiction, depression, personality disorder) were similar in pre- and post-sectorization groups.

**Table 2:** Sample characteristics: pre-reform and post-reform groups from the SMI-O sample

Variable	Pre-reform	Post-reform	Test of difference
N	451	198	
Female	53.66%	62.64%	χ^2^(df = 1) = 4.25, p < 0.05
Mean birthyear (SD)	1937 (11)	1937 (11)	
Diagnosis at First SMI-O Survey			χ^2^ (df = 5) = 126.55, p < 0.01
Drug addiction	2.56%	6.55%	
Psychosis	52.56%	30.33%	
Depression	22.16%	25.41%	
Neurosis	11.65%	26.23%	
Personality disorder	6.53%	6.56%	
Other	4.54%	4.92%	
Civil status at age 65			χ^2^ (df = 3) = 10.54, p < 0.05
Married	15.36%	23.48%	
Never married	46.43%	34.78%	
Divorced	33.21%	30.43%	
Widowed	5.00%	11.30%	
Education			χ^2^ (df = 6) = 3.01, *ns*
Less than 9 years	45.39%	41.48%	
9 years	12.53%	13.07%	
Up to 2 years secondary education	26.48%	25.57%	
3 years secondary education	4.73%	6.82%	
Less than 3 years post-secondary	6.86%	7.39%	
More than 3 years post-secondary	3.78%	5.68%	
Post-graduate education	0.24%	0.00%	
Mean year of first institutionalization (SD)	1975.9 (5.74)	1992.8 (6.99)	t(647) = 32.44, p < 0.01
Mean age at first institutionalization (SD)	38.46 (11.92)	54.55 (12.30)	t(647) = 15.24, p < 0.01
Mean number of stays at institution (SD)	47.96 (46.16)	44.74 (47.04)	t(647) = 0.82, *ns*
Mean total years institutionalization (SD)	4.26 (6.97)	0.48 (0.62)	t(647) = 7.64, p < 0.01
Mean length of stay: days (SD)	99.37 (329.4)	10.59 (23.79)	t(647) = 3.80, p < 0.01
Mean age at first SMI-O survey (SD)	61.83 (11.27)	63.45 (11.25)	t(647) = 1.68, *ns*

**Fig. 2:**
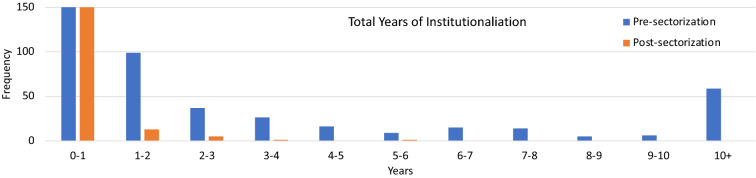
Comparison of categorical variable of total years of institutionalization across pre-sectorization and post-sectorization groups

With regard to the three-time metrics, group membership (pre- or post-sectorization) captured the year of first entry into a mental hospital. Second, the pre-sectorization group was institutionalized at an earlier age, on average, and thus experienced longer age stay in a mental hospital. Because the length of institutionalization was not normally distributed, we transformed the variable into a categorical variable by determining the number of people who experienced up to 1 year, 2 years, 3 years, etc., of institutionalization. Results are presented in Fig. 2 and comparing frequencies across pre- and post-sectorization groups indicated a significant difference [chi-square (df = 10) = 160.84, *p* < 0.01]. In fact, 31.6% of the pre-sectorization group had accumulated more than 10 years in a mental hospital (maximum institutionalization = 57.5 years), whereas no one in the post-sectorization group had accumulated more than 6 years in a mental hospital. Third, the longitudinal follow-up allowed for investigation of changes resulting from aging with SMI, as demonstrated using categorical and continuous approaches. Note that there was not a significant difference between groups in mean number of stays at an institution, only in the average length of each stay in hospital (99 days vs 10 days).

#### Categorical data analyses

**Fig. 3:**
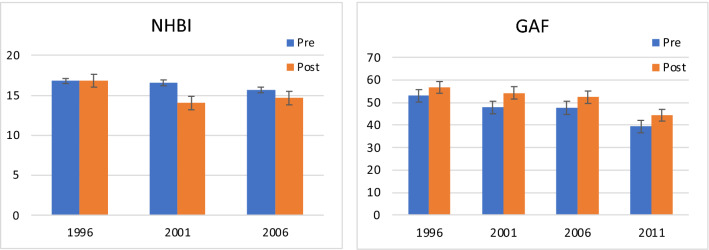
Pre-sectorization and post-sectorization group mean differences in NHBI and GAF measures of functioning at each survey year

Pre- and post-sectorization groups were compared on the three functional outcomes measures at each survey wave. Results for the NHBI and GAF measures are presented in Fig. 3. Although the two groups started with the same NHBI value in 1996, the pre-sectorization group experienced more functional difficulties as measured by the NHBI, a difference that was significant in 2001 [t(238) = 2.81, p < 0.01]. Results for the GAF were similar: the two groups started out with nearly identical means in 1996 but the pre-sectorization group demonstrated reduced functioning compared to the post-sectorization group at subsequent surveys. The differences between groups were significant in 2001 [t(241) = 3.08, p < 0.01] and 2006 [t(152) = 2.04, p < 0.05]. Although the difference between the two groups was of the same magnitude in 2011, the difference did not achieve statistical significance due to the reduction in sample size.

**Fig. 4:**
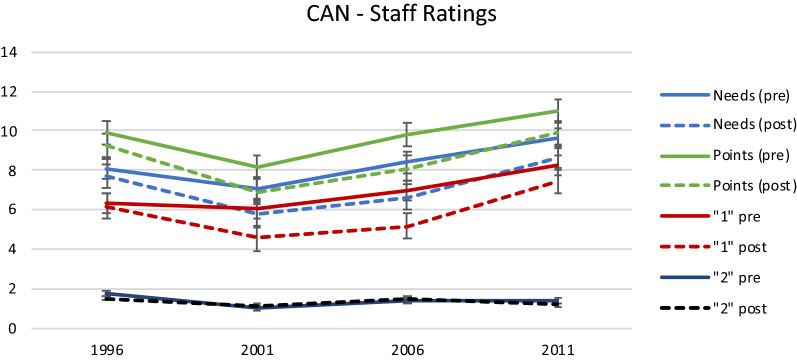
Pre-sectorization and post-sectorization group mean differences in CAN measures of functioning as rated by the staff at each survey year

Because clients had a tendency to rate their performance higher than staff [23], analysis of the CAN focused on staff ratings of functioning and results are presented in Fig. 4. Similar to the results for the NHBI and GAF, the two groups had fairly similar levels of functioning in 1996 but at subsequent surveys the pre-sectorization group was rated as having more need, more points, and more moderate problems (“1” ratings). There were no group differences at any survey in severe problems (“2” ratings). Differences between the two groups on needs, points, and “1” ratings were significantly different in 2001 and 2006. Again, although the differences between the two groups was of the same magnitude in 2011 on needs and points, the differences did not achieve statistical significance due to the reduction in sample size. Increasing mean needs and points in both groups from 2001 and 2011 likely reflect the impact of the aging process.

#### Continuous data analyses

Results of predicting functional outcome measures from group membership (pre- and post-sectorization), total years of institutionalization, age at first SMI-O survey, sex, and psychosis diagnosis (no or yes) are presented in Table 3. As the F-statistics indicate, all but one regression explained a significant proportion of the variance in the outcome variable; the exception was for the Points variable from the CAN in 2011. Adjusted R^2^ values, indicating the proportion of variance explained, ranged from 0.05 to 0.41 with a median value of 0.17. Regression results were fairly consistent across outcomes variables. In each regression, the time metric of total years of institutionalization was a significant predictor of the outcome variable, even when age, sex, and psychosis diagnosis were taken into account. In contrast, the time metric of group membership (pre- and post-sectorization) did not contribute significantly to the prediction of outcome variables. Finally, the time metric of age contributed significantly to the prediction of outcome variables about half the regression analyses. For the GAF, psychosis diagnosis was a consistent predictor of functioning at each survey. However, psychosis diagnosis never contributed to the prediction of functioning as measured by the CAN; total years of institutionalization was the only consistent predictor of CAN outcomes. Sex was never a significant predictor. Subsequent analyses investigating the role of interaction terms (e.g., sex x diagnosis) did not provide any evidence to support a significant role for interaction terms in the regression analyses.

**Table 3:** Results of regression analyses predicting outcomes variables

Variable	Parameter Estimates (SE)					F	df	Adjusted R^2^
	Total years institutionalization	Pre-reform = 1, Post-reform = 2	Age at first SMI-O survey	Sex 0 = male, 1 = female	Psychosis 0 = no, 1 = yes			
NHBI 1996	0.18 (0.04)**	0.46 (0.69)	0.23 (0.02)**	− 0.56 (0.54)	− 0.31 (0.53)	35.17**	(5, 296)	0.36
NHBI 2001	0.22 (0.04)**	− 1.15 (0.77)	0.25 (0.03)**	− 0.33 (0.61)	− 0.39 (0.62)	32.22 **	(5, 223)	0.41
NHBI 2006	0.33 (0.09)**	− 0.29 (1.05)	0.26 (0.06)**	− 0.93 (0.84)	− 0.61 (0.87)	7.82**	(5, 148)	0.18
GAF 1996	− 0.36 (0.11)**	− 0.02 (2.17)	0.06 (0.07)	1.67 (1.68)	− 5.78 (1.66)**	7.46**	(5, 296)	0.10
GAF 2001	− 0.52 (0.13)**	2.58 (2.24)	− 0.20 (0.08)*	0.10 (1.76)	− 2.79 (1.80)	9.45**	(5, 226)	0.15
GAF 2006	− 0.76 (0.21)**	0.86 (2.42)	− 0.09 (0.14)	1.67 (1.94)	− 4.67 (2.01)*	5.79**	(5, 147)	0.14
GAF 2011	− 1.63 (0.39)**	1.87 (3.41)	0.28 (0.21)	− 0.75 (2.81)	− 6.89 (2.76)*	6.00**	(5, 75)	0.24
CAN								
Needs 1996	0.13 (0.03)**	0.14 (0.50)	0.06 (0.02)**	− 0.44 (0.39)	− 0.29 (0.39)	10.27**	(5, 283)	0.14
Needs 2001	0.15 (0.03)**	− 0.67 (0.50)	0.05 (0.02)*	− 0.24 (0.40)	− 0.08 (0.40)	11.85**	(5, 226)	0.19
Needs 2006	0.24 (0.06)**	− 0.66 (0.70)	0.08 (0.04)*	− 0.45 (0.56)	0.71 (0.57)	7.07**	(5, 141)	0.17
Needs 2011	0.41 (0.10)**	0.11 (0.88)	0.03 (0.06)	− 0.06 (0.74)	− 0.22 (0.73)	3.27**	(5, 77)	0.12
Points 1996	0.13 (0.04)**	− 0.15 (0.71)	0.03 (0.02)	− 0.48 (0.56)	− 0.41 (0.55)	4.25**	(5, 283)	0.05
Points 2001	0.15 (0.04)**	− 0.59 (0.65)	0.03 (0.02)	− 0.44 (0.51)	0.08 (0.52)	6.37**	(5, 226)	0.10
Points 2006	0.27 (0.07)**	− 0.71 (0.88)	0.04 (0.05)	− 0.69 (0.71)	0.70 (0.72)	4.95**	(5, 141)	0.12
Points 2011	0.36 (0.13)**	− 0.18 (1.11)	− 0.03 (0.07)	− 0.42 (0.93)	− 0.31 (0.91)	1.79	(5, 77)	0.05
“1” in 1996	0.13 (0.02)**	0.43 (0.46)	0.08 (0.01)**	− 0.40 (0.36)	− 0.16 (0.36)	15.65**	(5, 283)	0.20
“1” in 2001	0.16 (0.03)**	− 0.73 (0.47)	0.06 (0.02)**	− 0.05 (0.38)	− 0.25 (0.38)	14.96**	(5, 226)	0.23
“1” in 2006	0.22 (0.05)**	− 0.60 (0.63)	0.11 (0.03)**	− 0.22 (0.51)	0.72 (0.52)	8.48**	(5, 141)	0.20
“1” in 2011	0.46 (0.10)**	0.39 (0.88)	0.09 (0.06)	0.30 (0.74)	− 0.13 (0.72)	4.58	(5, 77)	0.18

^*^p < .05

^**^p < .01

## Discussion

Investigation of the reorganization of psychiatric care and functioning and needs indicated that results varied depending on the time metric considered: (a) year of first entry into a mental hospital, (b) total years of institutionalization, or (c) changes resulting from aging with SMI. Aging contributed to prediction of functioning for about half the outcome variables. However, the most important time metric was length of stay in a mental hospital, which predicted outcomes in every case even when psychosis diagnosis was taken into account. Moreover, the pre-sectorization group was much more likely to have experienced extensive time in a mental hospital. All in all, compared to the post-sectorization group, the pre-sectorization group were found to be disadvantaged in that their level of functioning was lower, and they had more unmet needs.

The fact that the two groups are identical in terms of both the average year of birth (1937) and birth year range (1910–1951) means that the post-sectorization group was significantly older when first admitted at the psychiatric clinic (average 54 years old), compared to the pre-sectorization group who on average was 38 years old at the time of first admission. This means that the studied groups belong to the same generation in terms of age, at the same time as they constitute two completely different “patient generations” in relation to psychiatry care ideology and organization of psychiatric care. Based on the development of psychiatric care, the pre-group belongs to a “traditional generation” of patients who in many cases encountered a form of care that to varying degrees was characterized by what Goffman [29] called the total institution, which was both a place meant to separate patients from society and to provide treatment (30; cf. Author). The care provided at the mental hospitals included all aspects of a person's life, both material and social welfare—no activity at the mental hospital was carried out without being thought of as part of the treatment (Author). According to the mental hospital care ideology, work, leisure activities, housing, spiritual experience, etc., were part of the patient's treatment.

The post-sectorization group, on the other hand, belongs to a “modern patient generation” in relation to psychiatric care, despite the fact that they are part of the same generation as the pre-sectorization group in terms of age. That the post-sectorization were significantly older at the time for first being admitted to psychiatric inpatient care probably also meant more opportunities and higher probabilities for being established in society. We found no differences concerning education or source of income. However, the fact that the post-sectorization group significantly more often were married (23%), or a widow/-er (11%) compared to the pre-sectorization group (15% and 5% respectively) indicate that the post-sectorized group might have had a more stable and securing family life and when comparing living conditions. The post-sectorization group had shorter accumulated time of hospitalization but with no differences in number of stays. This meant that the long-stay hospitalization was not replaced by frequent admissions, so called revolving door-patients [31]. The new care ideology, which the post-sectorization group met when they were first admitted to psychiatric inpatient care, was characterized by optimism among the staff and an explicit effort to avoid hospitalization [32] as well as maintain continuity in the patient-staff relationship.

Previous research has argued that people with SMI did not benefit from the outpatient care as part of the sectorization [11]. However, results from our analyses, showing the post-sectorization group to have stays in inpatient one-tenth as long as the pre-sectorization group, on average, might indicate the opposite. That is, the post-sectorization group more likely benefited from open psychiatric care before, after, or in between periods of hospitalization. At least two organizational changes might work as explanations. First, at the time of the post-sectorization group’s first admission, the new care ideology pleaded for outpatient care instead of hospitalization as far as possible [17, 32, 33]. Secondly, the reduction in the number of beds, as part of the ongoing process of the reorganization of psychiatric care probably meant greater resistance to the admission of patients without a previous history of hospitalization [17, 34, 35], while the availability of outpatient care speaks in favour of their use.

The probability of experiencing more time in mental hospital during the era of a care ideology characterized as that of the total institution, not only meant longer periods of admission but also being at greater risk to develop institutional syndrome, which Bean and Mounser [36] present from Barton’s [37] and Goffman’s [29] work. This resembles the Swedish term “hospitaliseringsskador” [16] which captures the idea of being harmed or changed by medical or psychiatric care, such as the psychological changes that result from long-term stay in any institutional setting. Patients with long-term stay in a mental hospital will develop a “hospital mentality”: apathy, lack of initiative, loss of interest, lack of individuality, submissiveness, reduced motor function, and loss of ability for long-term planning. This might partly explain why the pre-sectorization group was rated by staff as having lower social, occupational, and psychological functioning (GAF), and in need of more support, while growing older, compared to the post-sectorization group. It is likely that the experience of extensive time in a mental hospital, besides being stigmatising, worked as a double jeopardy: aging and a long history of institutionalization combined to create poorer outcomes. The concept of double jeopardy has been used to examine the interaction of gender, race, socioeconomic position, as well as diabetes mellitus, with mental health care and outcomes (e.g. [38–41] but this study may represent the first attempt to examine how aging and long-term institutionalization interact to affect outcomes. Differences between the pre-sectorization group and the post-sectorization group increased during the follow-up study 1996–2011 with regard to satisfied and unsatisfied needs and global functions.

Given that time in a mental hospital is the most important predictor of functional level and the number of unmet needs, and that first admission to a mental hospital prior to the implementation of the sectorization resulted in significantly longer hospital stays, it means that the sectorization of psychiatric care to a large extent also had a positive effect on the group with SMI, and thus not only did the “worried well” benefit as previously described [9, 10]. In other words, in our study the “modern generation” of patients with SMI did benefit from the more open and society-based psychiatry and thereby did benefit from the sectorized psychiatry. The reform of psychiatric care in the studied municipality meant in parallel both an expansion of outpatient care in various places in the municipality and a gradual reduction of care places in the large old mental hospital. One possible explanation for the differences between the two groups examined is that the post-sectorization group probably also received psychiatric treatment in outpatient care, while many in the pre-sectorization group to a greater extent remained in the mental hospital.

### Strengths and limitations

The strength of the article is that we collected data at four different times, five years apart for a total of 15 years of follow-up. We have also incorporated data from various national registers with data beginning from the early 1900s. Limitations in the study, which can of course create bias in the interpretation, is that we do not have information about different degrees of severity of the diseases, nor do we have information about treatment over time. For example, there were no effective drug treatments for severe mental illness until the mid-1950s, and since then the development of psychotropic drugs has been rapid. How this development has affected the possibilities for reduced inpatient care, and hence, increased outpatient care, we can only speculate about. On the other hand, at the time of the sectorization in 1985, treatment with psychotropic drugs, as well as various psychotherapeutic treatments, were common and not essentially different from today's treatments.

## Conclusions

Sectorization thus turned out to be an important divide which could explain differences in two groups of the same age but with different institutional history: “modern” and “traditional” patient generations that received radically different types of care. The results indicate that the sectorization of psychiatric care might be as important in the studied municipality as the Mental Health Care Reform of 1995, although a relatively quiet revolution [8].

## Data Availability

All data are in a secured file at the university.

## Abbreviations

SMI: Severe mental illness
SMI-O: People who in this study in year 2016 were 65 years or older: that is severely mental ill-older
NBHWI: The National Board of Health and Welfare’s Inventory form
GAF: General Assessment of Functioning
CAN: Camberwell Assessment of Need

## References

[1] Leff J, Trieman N, Knapp M, Hallam A (2000). The TAPS project: a report on 13 years of research, 1985–1998. Psychiatr Bull.

[2] Scull AT (1984). Decarceration—community treatment and the deviant—a radical view.

[3] Craig T (1998). Homelessness and mental health. Psychiatr Bull.

[4] Modestin J, Ammann R (1995). Mental disorders and criminal behaviour. Br J Psychiatry.

[5] Mortensen PB, Juel K (1993). Mortality and causes of death in first admitted schizophrenic patients. Br J Psychiatry.

[6] Warner R (2013). Recovery from schizophrenia: psychiatry and political economy.

[7] Leff J, Trieman N (2000). Long-stay patients discharged from psychiatric hospitals: social and clinical outcomes after five years in the community. The TAPS Project 46. Br J Psychiatry..

[8] Forslund T, Kosidou K, Wicks S, Dalman C (2020). Trends in psychiatric diagnoses, medications and psychological therapies in a large Swedish region: a population-based study. BMC Psychiatry.

[9] Gittelman M (1972). Sectorization: the quiet revolution in European mental health care. Am J Orthopsychiatry.

[10] Lindholm H (1983). Sectorized psychiatry. A methodological study of the effects of reorganization on patients treated at a mental hospital. Acta Psychiatr Scand Suppl.

[11] Stefansson CG, Hansson L (2001). Mental health care reform in Sweden, 1995. Acta Psychiatr Scand.

[12] Becker T, Vázquez-Barquero JL (2001). The European perspective of psychiatric reform. Acta Psychiatr Scand.

[13] Berggren B, Cullberg J. Psykiatri i omvandling. SPRI, editor. 1978.

[14] Gittelman M (2005). The neglected disaster. Int J Ment Health.

[15] Henckes N, Kritsotaki D, Long V, Smith M (2016). French deinstitutionalisation or the irony of success: psychiatrists, the state and the transformation of the French psychiatric system. Deinstitutionalisation and after.

[16] Socialstyrelsen. Psykiatrisk vård utan mentalsjukhus [psychiatric care without mental hospitals]. Socialstyrelsen redovisar 1982;21982.

[17] Socialstyrelsen. Riktlinjer för 80-talets psykiatriska vård [guidelines for psychiatric care in the 80s]. Socialstyrelsen anser 1980;21980.

[18] Wagner PJ, Curran P (1984). Health beliefs and physician identified "worried well". Health Psychol.

[19] Prop. Psykiskt stördas villkor [the condition of the psychiatric disordered]. Stockholm1993/94:218.

[20] Socialdepartementet. Välfärd och valfrihet: service, stöd och vård för psykiskt störda: slutbetänkande av Psykiatriutredningen [Welfare and freedom of choice: service, support and care for psychiatric disordered: final report]. Stockholm: Allmänna förlaget; 1992.

[21] Arvidsson H (2008). The development of needs in a group of severely mentally ill. Soc Psychiatry Psychiatr Epidemiol.

[22] Severely AH (1995). Severely and persistently mentally ill: a changing group. Ten years after the 1995 Swedish mental health care reform. Nord J Psychiatry.

[23] Finkel D, Bülow PH, Wilińska M, Jegermalm M, Torgé CJ, Ernsth Bravell M (2021). Does the length of institutionalization matter? Longitudinal follow-up of persons with severe mental illness 65 years and older: shorter-stay versus longer-stay. Int J Geriatr Psychiatry.

[24] Arvidsson H. After the 1995 Swedish Mental Health Care Reform-a follow-up study of a group of severely mentally ill. 2004. 10.1007/s00127-008-0356-7 18438596

[25] Frances A, Pincus H, First M. The global assessment of functioning scale (GAF). Diagnostic and statistical manual of mental disorders. 1994. p. 4.

[26] Startup M, Jackson MC, Bendix S (2002). The concurrent validity of the Global Assessment of Functioning (GAF). Br J Clin Psychol.

[27] Phelan M, Slade M, Thornicroft G, Dunn G, Holloway F, Wykes T (1995). The Camberwell Assessment of Need: the validity and reliability of an instrument to assess the needs of people with severe mental illness. Br J Psychiatry.

[28] Reynolds T, Thornicroft G, Abas M, Woods B, Hoe J, Leese M (2000). Camberwell Assessment of Need for the Elderly (CANE): development, validity and reliability. Br J Psychiatry.

[29] Goffman E (1961). Asylums: essays on the social situation of mental patients and other inmates.

[30] Brunt D (2002). Supported housing in the community for persons with severe mental illness.

[31] Haywood TW, Kravitz HM, Grossman LS, Cavanaugh JL, Davis JM, Lewis DA (1995). Predicting the “revolving door” phenomenon among patients with schizophrenic, schizoaffective, and affective disorders. Am J Psychiatry.

[32] Bachrach LL, Knudsen HC, Thornicroft G (1996). Deinstitutionalisation: promises, problems and prospects. Mental health service evaluation.

[33] Bachrach LL, Leff J (1997). Lessons from the American experience in providing community-based services. Care in the community Illusion or reality?.

[34] Ramon S (1996). Mental health in Europe. Ends, beginnings and rediscoveries.

[35] Payne S, Bartlett P, Wright D (1999). Outside the walls of the asylum? Psychiatric treatment in the 1980s and 1990s. Outside the walls of the asylums.

[36] Bean P, Mounser P (1993). Discharged from mental hospitals.

[37] Barton R (1959). Institutional neurosis.

[38] Marston EG, Russell MA, Obsuth I, Watson GK, Miller S, Leve LD, Kerig PK (2012). Dealing with double jeopardy: mental health disorders among girls in the juvenile justice system. Delinquent girls: contexts, relationships, and adaptation.

[39] Das-Munshi J, Stewart R, Morgan C, Nazroo J, Thornicroft G, Prince M (2016). Reviving the ‘double jeopardy’hypothesis: physical health inequalities, ethnicity and severe mental illness. Br J Psychiatry.

[40] Mendelson T, Kubzansky LD, Datta GD, Buka SL (2008). Relation of female gender and low socioeconomic status to internalizing symptoms among adolescents: a case of double jeopardy?. Soc Sci Med.

[41] Blanchard E, Samaras K (2014). Double jeopardy: diabetes and severe mental illness. Addressing the special needs of this vulnerable group. Diabetes Manag.

